# Can CCRT/RT Achieve Favorable Oncologic Outcome in Rectal Cancer Patients With High Risk Feature After Local Excision?

**DOI:** 10.3389/fonc.2022.767838

**Published:** 2022-03-23

**Authors:** Seijong Kim, Jung Wook Huh, Woo Yong Lee, Seong Hyeon Yun, Hee Cheol Kim, Yong Beom Cho, Yoon Ah Park, Jung Kyong Shin

**Affiliations:** Department of Surgery, Samsung Medical Center, Sungkyunkwan University School of Medicine, Seoul, South Korea

**Keywords:** rectal cancer, local excision, neoadjuvant chemoradiotherapy, recurrence, survival

## Abstract

**Purpose:**

The oncologic outcome of concurrent chemoradiotherapy (CCRT) after local excision in patients with high-risk early rectal cancer as compared with radical operation has not been reported. The aim of this study is to compare the oncologic outcome between radical operation and adjuvant CCRT after local excision for high-risk early rectal cancer.

**Materials and Methods:**

From January 2005 to December 2015, 266 patients diagnosed with early rectal cancer and treated with local excision who showed high-risk characteristics were retrospectively analyzed. Propensity score matching was applied in a ratio of 1:4, comparing the CCRT/radiotherapy (RT) (n = 34) and radical operation (n = 91) groups. Univariate and multivariate analyses were performed to identify prognostic factors for survival.

**Results:**

The median follow-up period was 112 months. The 5-year disease-free survival rate and the 5-year overall survival of the radical operation group were significantly higher than those of the CCRT/RT group after propensity score matching (96.7% vs. 70.6%, *p <*0.001; 100% vs. 91.2%, *p* = 0.005, respectively). In a multivariate analysis, salvage therapy type and preoperative carcinoembryonic antigen (CEA) were prognostic factors for 5-year disease-free survival (*p <*0.001 and *p* = 0.021, respectively). The type of salvage therapy, the preoperative CEA, and the pT were prognostic factors for 5-year overall survival (*p* = 0.009, *p* = 0.024, and *p* = 0.046, respectively).

**Conclusions:**

Patients who undergo radical operations after local excision with a high-risk early rectal cancer had better survival than those treated with adjuvant CCRT/RT. Therefore, radical surgery may be recommended to high-risk early rectal cancer patients who have undergone local excision for more favorable oncologic outcomes.

## Introduction

Rectal cancer is one of the most common types of cancers in humans for which radical resection is the standard treatment for rectal cancer (1). However, radical surgery, such as low anterior resection (LAR) and abdominoperineal resection (APR), is associated with significant morbidities such as anastomosis site leakage, sexual dysfunction, and urinary dysfunction. In addition, some patients require permanent colostomy after APR, leading to poor quality of life (1, 2). Therefore, local excision has been used as an alternative surgical option in early rectal cancer (3). Local excision in the appropriate patients can minimize morbidity and mortality of radical operation and provide long-term survival (3, 4). However, local excision of rectal cancer of high-risk pT1 or pT2 has a higher risk of recurrence than radical operation (1, 5). The National Comprehensive Cancer Network guideline suggests radical surgery or chemoradiotherapy as adjuvant therapy options for rectal cancer treated with local excision for those with pT1 and high-risk features or pT2 (6). Many studies on the effectiveness of adjuvant concurrent chemoradiotherapy (CCRT) following local excision have shown favorable oncologic outcomes compared with local excision alone in early rectal cancer with high-risk characteristics (7–9). However, studies comparing oncologic outcomes between adjuvant CCRT and radical operation have not yet been reported. Therefore, the objective of this study was to determine oncologic outcomes between radical operation and adjuvant CCRT after local excision for high-risk early rectal cancer.

## Materials and Methods

This retrospective study enrolled all patients with early rectal cancer without lymph node metastasis who were treated with local excision with high-risk pathologic results at the Samsung Medical Center between January 2005 and December 2015. Exclusion criteria were as follows: recurrent disease, palliative surgery, preoperative chemoradiation therapy, familial cancer, or lack of follow-up data. The preoperative diagnosis was made with a digital rectal examination, colonoscopy, and MRI. Local excision included endoscopic mucosal resection, endoscopic submucosal dissection, polypectomy, transanal excision, and transanal endoscopic microsurgery. Lymph nodal status was defined by abdominopelvic CT or rectum MRI.

The high-risk group included tumor size ≥3 cm, positive resection margin, and unfavorable tumor characteristics such as lymphovascular invasion (LVI), poor differentiation, mucinous adenocarcinoma, signet ring cell type, deeper than SM1, and pathologic T2 rectal cancer (10, 11).

The patients were divided into two groups: 1) those who were treated with CCRT/radiotherapy (RT) after local excision and 2) those who were treated with radical operation after local excision. The radical operation group of 218 patients underwent a standard operation with total mesorectal excision. The adjuvant therapy group consisted of 48 patients who experienced CCRT or RT, namely, 10 treated with RT and 38 treated with CCRT. A total of 34 patients refused radical operation, although surgeons fully explained the risk–benefit of CCRT. In addition, three patients underwent CCRT/RT because they needed APR due to tumor location, which is a destructive operation. One patient was old with several underlying diseases, indicating a lack of suitability for general anesthesia. This patient was treated with CCRT. Ten patients had no listed reason for avoiding surgery. Regarding the chemotherapy regimen, 5-fluorouracil was administered to 20 patients, and Xeloda was administered to 11 patients, while both 5-fluorouracil and leucovorin were administered to three patients. Three patients did not have records of the chemotherapy regimen. All patients received 45 to 60 Gy of radiation delivered in 4.5–6 weeks. This study was approved by the Institutional Review Board of the Samsung Medical Center.

These patients were followed up at 3-month intervals for the first 2 years after surgery, at 6-month intervals for the next 3 years, and then once a year. The follow-up included physical examination, chest radiography or CT, serum carcinoembryonic antigen (CEA) level, and abdominopelvic CT. Colonoscopy was examined 1 year after the operation and then every other year. Patients suspected of recurrence were examined during routine follow-up and subjected to rectum MRI for inspection.

### Statistical Analysis

All statistical analyses were performed using SAS version 9.4 (SAS Institute, Cary, NC, USA) and R 4.0.0 (Vienna, Austria; http://www.R-project.org/). Categorical variables were analyzed with Chi-square and Fisher’s exact tests. Continuous variables were analyzed with t-test and Wilcoxon rank sum test. Cox proportional hazards model was used to analyze risk factors that affected disease-free survival and overall survival. Variables that were associated with p-value <0.1 on univariate analysis were included in multivariate analysis in DFS. Survival curves were designed using the Kaplan–Meier method. Differences between curves were evaluated using the log-rank test. Propensity-score matching (PSM) was used to balance the distribution of baseline pathologic variables and evaluate treatment efficacy between CCRT/RT and radical operation. Case matching was performed using propensity score of lymphovascular invasion, resection margin, gross type, tumor size, and depth of tumor. A 1:4 nearest neighbor matching without replacement was used. Patients treated with CCRT/RT were matched to radical operation patients with the closest estimated propensity scores. In selecting subjects for PSM, if there was no similar subject in the caliper we set up, the program did not select subjects by 1:4. Thus, there could be some cases not selected by four multiples. After matching, the fidelity of the model was tested. No covariate had a standardized mean difference >0.1 between the two groups. The average absolute standardized mean difference of covariates was 0.040. A p-value <0.05 was considered statistically significant.

## Results

A total of 266 patients with early rectal cancer who underwent local excision and had high-risk factors were analyzed. This analysis included 165 (62.0%) men and 101 (38.0%) women with a median age of 58 years (range, 33–84 years). The median tumor diameter was 1 cm (range, 0.1–8 cm). The median distance from the anal verge to cancer was 7 cm (range, 1–12 cm). The numbers of patients with LVI, tumor budding, positive resection margin, and tumor depth deeper than SM1 and pT2 were 81 (30.5%), 33 (24.8%), 61 (22.9%), 200 (75.2%), and 34 (12.8%), respectively. In pathological results, seven (2.6%) patients showed poor differentiation and mucinous adenocarcinoma and signet ring cell. Seventy-nine (37.1%) patients had macroscopic ulceration in the specimen. The type of local excision and the number of patients were as follows: polypectomy, n = 111; transanal endoscopic microsurgery, n = 72; endoscopic mucosal resection, n = 36; endoscopic submucosal dissection, n = 31; and transanal excision, n = 16.

**Figure 1:**
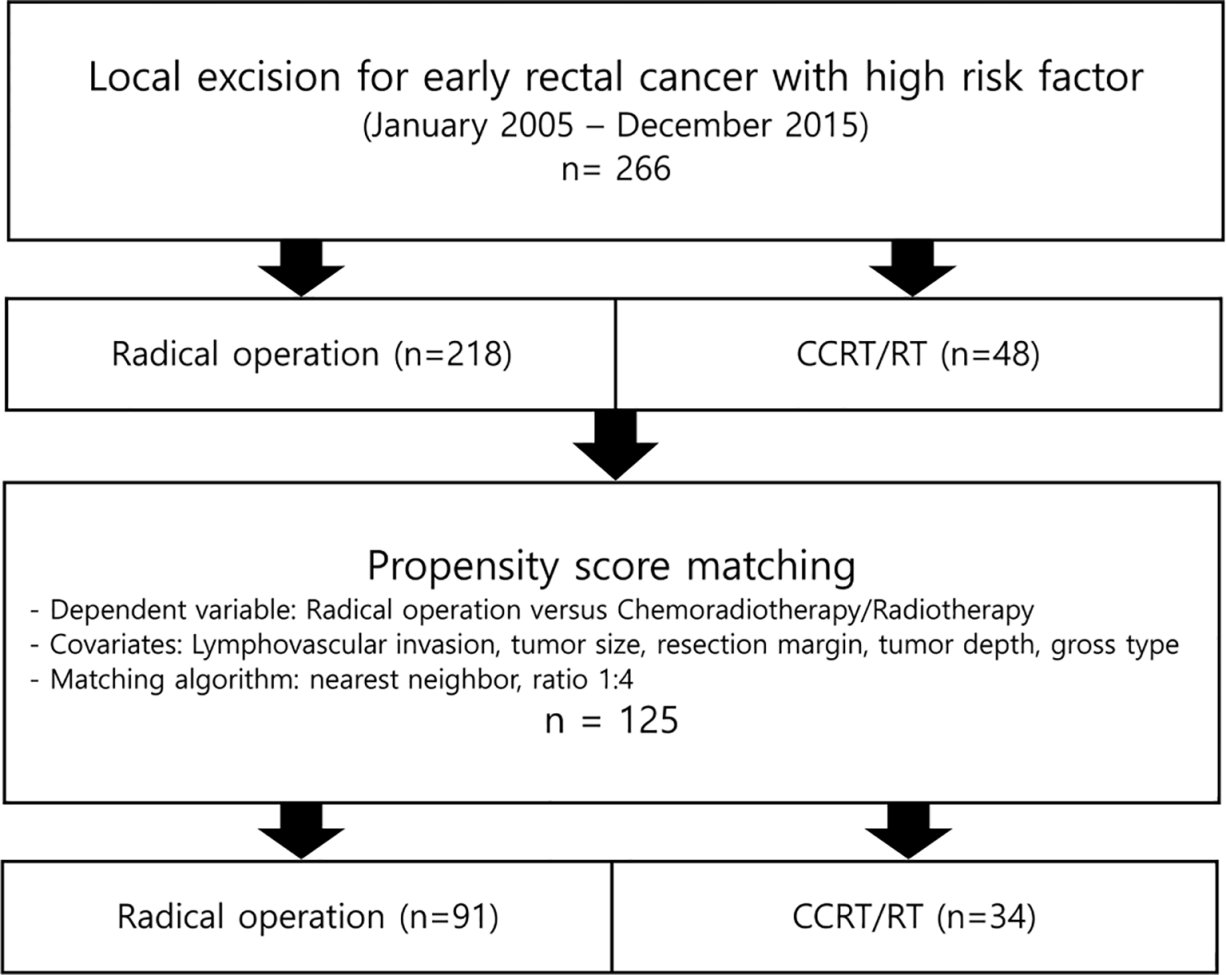
Flowchart showing study population selection and matching by propensity score.

The baseline characteristics of the patients before and after the matching are presented in **Table 1**. There were statistically significant imbalances between the radical operation group and the CCRT/RT group for LVI, resection margin, macroscopic ulceration, tumor size, tumor depth, tumor budding, and distance from the anal verge to cancer. The process of PSM is shown in **Figure 1**. After matching, there were no significant group differences in the baseline demographic or histopathologic variables except for the distance from the anal verge to cancer.

**Table 1:** Comparison of baseline characteristics between radical operation and chemoradiotherapy groups of patients (n = 266) before and after propensity score matching (n = 125).

	Before matching (n = 266)			After matching (n = 125)		
	Radical operation (n = 218)	CCRT/RT (n = 48)	*p-*Value	Radical operation (n = 91)	CCRT/RT (n = 34)	*p-*Value
Age, years			0.418			0.474
Median (range)	58 (33–84)	59.5 (34–83)		58 (37–84)	59 (34–83)	
Sex			0.941			0.392
Male	135 (61.9%)	30 (62.5%)		61 (67.0%)	20 (58.8%)	
Female	83 (38.1%)	18 (37.5%)		30 (33.0%)	14 (41.2%)	
BMI			0.551			0.974
Median (range)	24 (15.5–33)	23 (18–33)		23.7 (15–31)	23.6 (18–33)	
Preoperative CEA (ng/ml)			0.455			0.539
≤5	213 (97.7%)	47 (97.9%)		90 (98.9%)	34 (100%)	
>5	2 (0.9%)	1 (2.1%)		1 (1.1%)	0 (0%)	
Undescribed	3 (1.4%)	0 (0%)		0 (0%)	0 (0%)	
Tumor size (cm)			<0.001			0.245
Median (range)	1 (0.1–5)	1.8 (0.1–8)		1.57 (0.2–5)	1.90 (0.8–4.2)	
Distance from AV (cm)			<0.001			<0.001
Median (range)	7 (1–12)	4 (1–10)		7 (1–12)	4 (1–7)	
Gross type						0.604
No ulcer	100 (45.9%)	34 (70.8%)	<0.001	74 (81.3%)	29 (85.3%)	
Ulcer	74 (33.9%)	5 (10.4%)		17 (18.7%)	5 (14.7%)	
Undescribed	44 (20.2%)	9 (18.8%)		0 (0%)	0 (0%)	
Differentiation			0.113			0.809
Well-Moderate	214 (98.2%)	45 (93.8%)		89 (97.8%)	33 (97.1%)	
PD, MAC, SRC	4 (1.8%)	3 (6.2%)		2 (2.2%)	1 (2.9%)	
Resection margin			0.007			0.724
Negative	161 (73.9%)	44 (91.7%)		81 (89.0%)	31 (91.2%)	
Positive	57 (26.1%)	4 (8.3%)		10 (11.0%)	3 (8.8%)	
Tumor depth			0.283			0.128
SM1	57 (26.2%)	9 (18.8%)		22 (24.2%)	4 (11.8%)	
SM2, SM3, T2	161 (73.8%)	39 (81.2%)		69 (75.8%)	30 (88.2%)	
pT stage			0.020			0.592
T1	195 (89.5%)	37 (77.1%)		76 (83.5%)	27 (79.4%)	
T2	23 (10.5%)	11 (22.9%)		15 (16.5%)	7 (20.6%)	
Lymphovascular invasion			0.010			0.449
Negative	137 (62.8%)	21 (43.75%)		55 (60.4%)	18 (52.9%)	
Positive	57 (26.2%)	21 (43.75%)		36 (39.6%)	16 (47.1%)	
Undescribed	24 (11.0%)	6 (12.5%)		0 (0%)	0 (0%)	
Tumor budding			0.009			0.159
Negative	90 (41.3%)	10 (20.8%)		36 (39.5%)	8 (23.5%)	
Positive	23 (10.6%)	10 (20.8%)		16 (17.6%)	8 (23.5%)	
Undescribed	105 (48.1%)	28 (58.4%)		39 (42.9%)	18 (53.0%)	

BMI, body mass index; CEA, carcinoembryonic antigen; AV, anal verge; PD, poorly differentiated; MAC, Mucinous adenocarcinoma; SRC, signet ring cell.

The median time from local excision to radical operation was 28 days (range, 5–150 days). The median time from local excision to chemotherapy was 25 days (range, 12–90 days). The median time from local excision to RT was 26 days (range, 7–90 days). A total of 20 patients experienced recurrence after adjuvant treatment for local excision, namely, seven patients with local recurrence, 12 with systemic recurrence, and one with local and systemic recurrence. In patients with recurrence, nine had two high-risk factors, while 11 patients had only one.

There were 29 (13%) cases of postoperative morbidity in the radical operation group. In addition, 11 cases of leakage of the anastomosis site needed an operation for ileostomy. There was one case of rectovaginal fistula, five cases of postoperative bleeding, one case of intra-abdominal abscess, five cases of wound complication, and six cases of postoperative ileus.

### Oncologic Outcomes

**Figure 2:**
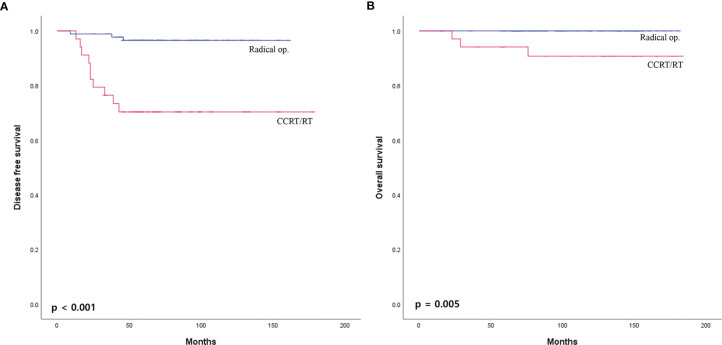
**(A)** Disease-free survival and **(B)** overall survival curves of patients with early rectal cancer between CCRT/RT and radical operation groups.

**Figure 3:**
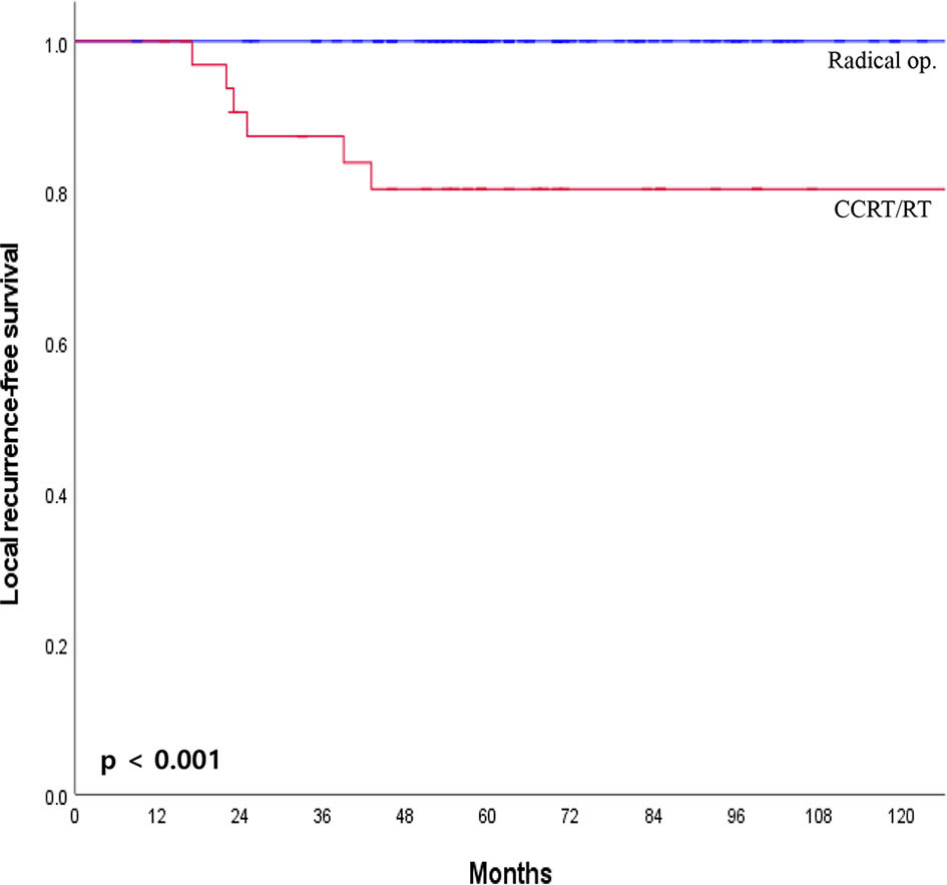
Local recurrence-free survival curves of patients with early rectal cancer between CCRT/RT and radical operation groups.

The median overall follow-up time was 112 months (range, 12–183 months). The survival outcome was analyzed in the group after PSM. Disease-free survival between the radical operation group and the CCRT/RT group was significantly different (96.7% vs. 70.6%, p <0.001; **Figure 2A**). The overall survival of the radical operation group was significantly higher than that of the CCRT/RT group (100% vs. 91.2%, p = 0.005; **Figure 2B**). In addition, local recurrence-free survival of the radical operation group was significantly higher than that of the CCRT/RT group (100% vs. 82.4%, p <0.001; **Figure 3**). Histopathologic characteristics of patients with local recurrence after local excision with adjuvant treatment are described in **Table 2**. Eight patients had local recurrence. All of these eight patients were treated with CCRT/RT after local excision. Local recurrence was developed in the rectum (four patients), perirectal lymph node (three patients), and pelvic lymph node (one patient). Of the eight patients diagnosed with local recurrence, six, one, and one underwent LAR, Hartmann’s operation, and LAR with pelvic lymph node dissection, respectively. One patient treated with LAR underwent APR due to local recurrence at the anastomosis site. Local recurrence was found again after APR. Four patients with local recurrence were diagnosed with systemic recurrence even after salvage operation and adjuvant chemotherapy. Systemic recurrence was found in 13 patients (six patients in the radical operation group and seven patients in the CCRT/RT group).

**Table 2:** Histopathologic characteristics of patients with local recurrence after local excision with adjuvant treatment.

Patient no.	Sex/Age	Differentiation	Tumor size	Depth of tumor	Resection margin	Lymphovascular invasion	Perineural invasion	Tumor budding
1	M/70	AC WD	1.6	SM2	Negative	Negative	Undescribed	Undescribed
2	M/63	AC PD	2.2	SM3	Negative	Negative	Undescribed	Undescribed
3	M/72	Mucinous AC	1.6	T2	Negative	Positive	Negative	Negative
4	M/36	AC MD	2.1	T2	Negative	Negative	Negative	Positive
5	M/62	Mucinous AC	1.5	SM2	Negative	Negative	Negative	Negative
6	M/40	AC MD	3	SM2	Negative	Positive	Negative	Negative
7	M/34	AC WD	1.2	SM2	Positive	Undescribed	Undescribed	Undescribed
8	F/63	AC MD	1.3	SM3	Negative	Positive	Negative	Negative

AC, adenocarcinoma; WD, well differentiated; PD, poorly differentiated; MD, moderately differentiated.

**Figure 4:**
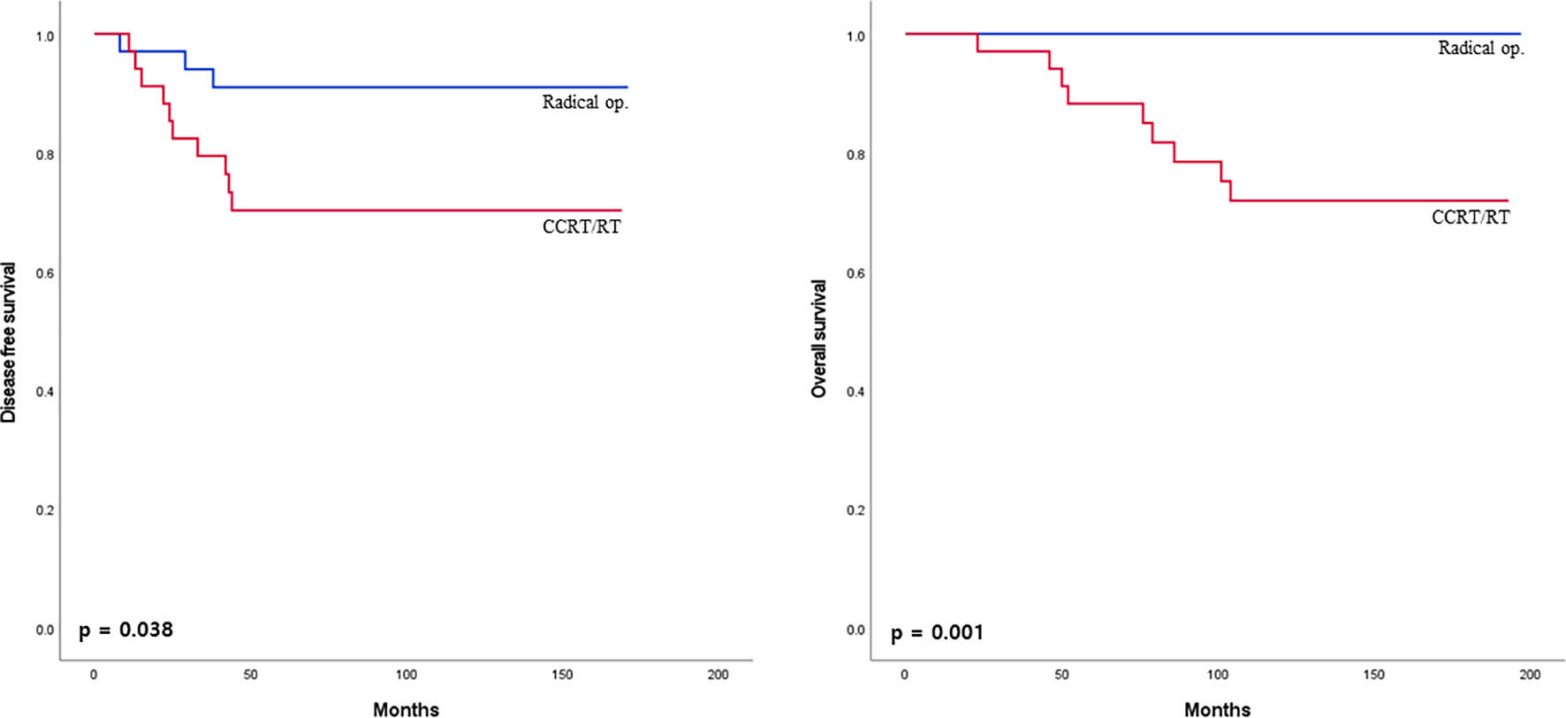
Disease-free survival curves of patients with early rectal cancer between CCRT/RT and radical operation groups in patient with mid to low rectal cancer.

The 5-year disease-free survival and overall survival between the radical operation and CCRT/RT groups were analyzed in mid to low rectal cancer within 10 cm from the anal verge. Five-year disease-free survival and overall survival rates were significantly higher in the radical operation group than in the CCRT/RT group (91.2% vs 70.6%. *p* = 0.038; 100% vs 73.5%, *p* = 0.001; **Figure 4**).

The prognostic factors for 5-year disease-free survival were salvage therapy type and preoperative CEA level in a multivariate analysis (p ≤0.001 and p = 0.021, respectively, **Table 3**). Multivariate analysis indicated that salvage therapy type, preoperative CEA level, and pT were independent prognostic factors for 5-year overall survival (p = 0.009, p = 0.024, and p = 0.046, respectively, **Table 4**).

**Table 3:** Univariate and multivariate analyses of prognostic factors for 5-year disease-free survival.

	Univariate analysis		Multivariate analysis		
	Survival (%)	*p-*Value	Hazard ratio	95% CI	*p-*Value
Salvage therapy type		<0.001	11.362	4.500–28.689	<0.001
Radical operation	96.8%				
CCRT/RT	70.8%				
Age, years		0.551			
Sex		0.369			
Male	91.0%				
Female	94.1%				
BMI (kg/m^2^)		0.366			
Preoperative CEA (ng/ml)		0.084	11.540	1.437–92.680	0.021
<5	92.3%				
≥5	66.7%				
Tumor size (cm)		0.290			
Gross type		0.391			
No ulcer	90.3%				
Ulcer	93.7%				
Differentiation		0.581			
Well-Moderate	92.3%				
PD, MAC, SRC	85.7%				
Resection margin		0.39			
Negative	91.7%				
Positive	93.4%				
Depth of tumor		0.959			
SM1	92.4%				
SM2, SM3, T2	92.0%				
Pathologic T stage		0.019	2.639	0.588–11.839	0.205
T1	93.5%				
T2	82.4%				
Lymphovascular invasion		0.036	0.914	0.169–4.945	0.917
Negative	93.5%				
Positive	88.9%				
Tumor budding		0.036	3.019	0.455–20.038	0.253
Negative	96.0%				
Positive	84.8%				

CCRT, concurrent chemoradiotherapy; RT, radiotherapy; BMI, body mass index; CEA, carcinoembryonic antigen; PD, poorly differentiated; MAC, Mucinous adenocarcinoma; SRC, signet ring cell.

**Table 4:** Univariate and multivariate analyses of prognostic factors for 5-year overall survival.

	Univariate analysis		Multivariate analysis		
	Survival (%)	*p-*Value	Hazard ratio	95% CI	*p-*Value
Salvage therapy type		0.004	12.12	1.829–80.298	0.009
Radical operation	99.5%				
CCRT/RT	89.6%				
Age, years		0.062			
Sex		0.811			
Male	97.6%				
Female	98.0%				
BMI (kg/m^2^)		0.719			
Preoperative CEA (ng/ml)		0.013	24.43	1.539–387.898	0.024
<5	100%				
≥5	97.7%				
Tumor size (cm)		0.180			
Gross type		0.930			
No ulcer	97.8%				
Ulcer	97.5%				
Differentiation		0.067			
Well-Moderate	98.1%				
PD, MAC, SRC	85.7%				
Resection margin		0.601			
Negative	97.1%				
Positive	100%				
Depth of tumor		0.630			
SM1	98.5%				
SM2, SM3, T2	97.5%				
Pathologic T stage		0.012	5.791	1.034–32.435	0.046
T1	98.7%				
T2	91.2%				
Lymphovascular invasion		0.031	3.181	0.537–18.840	0.202
Negative	99.5%				
Positive	93.8%				
Tumor budding		0.154			
Negative	100%				
Positive	93.9%				

CCRT, concurrent chemoradiotherapy; RT, radiotherapy; BMI, body mass index; CEA, carcinoembryonic antigen; PD, poorly differentiated; MAC, Mucinous adenocarcinoma; SRC, signet ring cell.

## Discussion

Early rectal cancer is defined as cancer limited to the submucosal region (T1) or muscularis propria (T2) without evidence of lymph node invasion (12, 13). The standard treatment for rectal cancer is radical surgery such as LAR or APR. However, radical surgery is associated with significant morbidity and mortality and may also lead to a permanent ostomy (1). In T1 rectal cancer, lymph node metastasis is observed in 0 to 11% of patients (14, 15). With a low risk of lymph node metastasis, local excision provides advantages over the radical operation, with lower morbidity and mortality. However, patients with unfavorable histopathological conditions after local excision, such as undifferentiated tumor, positive resection margin, LVI, tumor invasion deeper than SM1, or size larger than 3 cm, had a higher rate of recurrence than those after radical surgery (1, 16). Several studies have evaluated oncologic outcomes between adjuvant CCRT after local excision and local excision only in high-risk patients. However, studies that compare oncologic outcomes between adjuvant CCRT and radical operation in local excision patients with high-risk factors have not yet been reported. Our results showed that radical resection had excellent oncological outcomes compared with adjuvant CCRT/RT in 266 patients who underwent local excision with a median follow-up of 112 months.

The number of high-risk factors might be a risk factor for recurrence and even death. Saraste et al. have demonstrated that pT2, poor differentiation, and vascular infiltration are significant risk factors for lymph node metastases. In the presence of multiple risk factors, the potential risk of lymph node metastasis increases (17). Ueno et al. have shown that vascular invasion, unfavorable differentiation, and tumor budding are qualitative factors that can most effectively differentiate the risk of lymph node invasion. The higher the number of risk factors, the greater the involvement of the lymph nodes (18).

Local recurrence occurred in eight patients, and all patients were treated with adjuvant CCRT/RT. The efficacy of RT in locoregional recurrence remains controversial. Huh et al. have not shown local recurrence-free survival between CCRT and chemotherapy in stage III rectal cancer (19). Sauer et al. have shown that local control with postoperative CCRT produces a poor outcome than preoperative CCRT because tumor oxygenation is better with preoperative treatment than with postoperative treatment (20). Marijnen et al. have demonstrated that RT does not control local recurrence because RT typically leaves a positive resection margin (21). This poor local control effect of RT could be related to the higher local recurrence in the CCRT/RT group than in the radical operation group.

In this study, adjuvant CCRT/RT after local excision showed a poor oncologic outcome than radical operation. The 5-year disease-free survival and overall survivals were 70.6 and 91.2%, respectively, in the adjuvant CCRT/RT group, comparable with those of previous studies (7, 8, 11). The 5-year disease-free survival and overall survival in the radical operation group were 96.7 and 100%, respectively, similar to or higher than those of previous studies (1, 11, 16). Five-year disease-free survival in the radical operation group was similar to that of patients who underwent a radical operation for primary treatment (1, 16, 22).

In our study, salvage therapy type and preoperative CEA were prognostic factors for disease-free survival and overall survival. In addition, the pathologic T stage was a prognostic factor for overall survival. To support our research results, elevated preoperative CEA has previously been reported to be an independent prognostic factor for disease-free survival and overall survival in patients with rectal cancer (19, 23–25). Previous studies have also shown that the pathologic T stage is an independent prognostic factor for survival (26, 27). Compared with pT1, pT2 rectal cancer had an increased risk of lymph node metastasis. As the stage of pT increases, a poor prognosis is more likely (26, 28).

In the radical operation group, there were 29 (13%) cases of postoperative morbidity. In previous studies, postoperative complications after radical resection occurred in 14.6 to 48% of cases, significantly higher than the result of the present study (29). In addition, there was no mortality in this study cohort. However, no patient required permanent colostomy; 36 had a temporary ileostomy. This was due to the decision of the surgeon not to perform APR because it is a destructive operation. Patients who needed APR underwent CCRT/RT.

This study has several limitations. First, since it was conducted at a single center with retrospective nature, it was subject to several biases. We used PSM to overcome this limitation. Second, the regimens of chemoradiotherapy and RT were not standardized. For example, some patients underwent only RT without chemotherapy. This might have resulted in a difference in the treatment effect. Third, there was a lack of information on perineural invasion and tumor budding in pathologic reports after local excision, which could affect tumor recurrence. Fourth, due to various local excision methods such as full-thickness resection with transanal approach and endoscopic resection, there may be a bias affecting the oncologic outcome. Fifth, various endoscopists and surgeons performed local excision of the tumor, which could affect the pathologic result of local excision. Finally, in our study, the distance from the anal verge to the cancer was significantly shorter in the CCRT/RT group than in the radical surgery group. In most guidelines, RT has no therapeutic role in upper rectal cancer; therefore, we analyzed oncologic outcomes in mid to low rectal cancers below 10 cm from the anal verge. Five-year disease-free survival and overall survival were significantly higher in the radical surgery group than in the CCRT/RT group. Furthermore, we attempted to analyze the treatment role of CCRT/RT in low rectal cancer located below 5 cm from the anal verge; however, there was no statistically significant difference between the two groups due to the small number of patients. Further studies are needed to reevaluate and confirm these observations, allowing for better identification of high-risk early rectal cancer patients who may benefit from radiotherapy after undergoing local excision.

## Conclusion

We found that adjuvant CCRT/RT after local excision with high-risk factors had poorer oncologic outcomes than radical operation. Therefore, radical surgery may be recommended to high-risk early rectal cancer patients who have undergone local excision for more favorable oncologic outcomes.

## Data Availability Statement

The raw data supporting the conclusions of this article will be made available by the authors, without undue reservation.

## Ethics Statement

The studies involving human participants were reviewed and approved by the Institutional Review Board of Samsung Medical Center. Written informed consent for participation was not required for this study in accordance with the national legislation and the institutional requirements.

## Author Contributions

Conceptualization, SK, JWH, and WYL. Methodology, HCK and YAP. Software, JKS. Validation, JWH. Formal analysis, SK. Investigation, JKS. Resources, YBC and SHY. Writing—original draft preparation, JWH and SK. Writing—review and editing, SK, YAP, and SHY. Supervision, WYL. All authors listed have made a substantial, direct, and intellectual contribution to the work and approved it for publication.

## Conflict of Interest

The authors declare that the research was conducted in the absence of any commercial or financial relationships that could be construed as a potential conflict of interest.

## Publisher’s Note

All claims expressed in this article are solely those of the authors and do not necessarily represent those of their affiliated organizations, or those of the publisher, the editors and the reviewers. Any product that may be evaluated in this article, or claim that may be made by its manufacturer, is not guaranteed or endorsed by the publisher.
